# Modulation of the Tumor Microenvironment by Microbiota-Derived Short-Chain Fatty Acids: Impact in Colorectal Cancer Therapy

**DOI:** 10.3390/ijms24065069

**Published:** 2023-03-07

**Authors:** Sara Gomes, Ana Catarina Rodrigues, Valerio Pazienza, Ana Preto

**Affiliations:** 1 CBMA—Centre of Molecular and Environmental Biology, Department of Biology, University of Minho, 4710-057 Braga, Portugal; 2 IBS—Institute of Science and Innovation for Bio-Sustainability, University of Minho, 4710-057 Braga, Portugal; 3 CBMA/IBs/ARNET—Aquatic Research Network, University of Minho, 4710-057 Braga, Portugal; 4 Gastroenterology Unit, Fondazione IRCCS Casa Sollievo della Sofferenza Hospital, San Giovanni Rotondo, 71013 Foggia, Italy

**Keywords:** colorectal cancer (CRC), short-chain fatty acids (SCFAs), tumor microenvironment, therapy

## Abstract

Finding new therapeutic approaches towards colorectal cancer (CRC) is of increased relevance, as CRC is one of the most common cancers worldwide. CRC standard therapy includes surgery, chemotherapy, and radiotherapy, which may be used alone or in combination. The reported side effects and acquired resistance associated with these strategies lead to an increasing need to search for new therapies with better efficacy and less toxicity. Several studies have demonstrated the antitumorigenic properties of microbiota-derived short-chain fatty acids (SCFAs). The tumor microenvironment is composed by non-cellular components, microbiota, and a great diversity of cells, such as immune cells. The influence of SCFAs on the different constituents of the tumor microenvironment is an important issue that should be taken into consideration, and to the best of our knowledge there is a lack of reviews on this subject. The tumor microenvironment is not only closely related to the growth and development of CRC but also affects the treatment and prognosis of the patients. Immunotherapy has emerged as a new hope, but, in CRC, it was found that only a small percentage of patients benefit from this treatment being closely dependent on the genetic background of the tumors. The aim of this review was to perform an up-to-date critical literature review on current knowledge regarding the effects of microbiota-derived SCFAs in the tumor microenvironment, particularly in the context of CRC and its impact in CRC therapeutic strategies. SCFAs, namely acetate, butyrate, and propionate, have the ability to modulate the tumor microenvironment in distinct ways. SCFAs promote immune cell differentiation, downregulate the expression of pro-inflammatory mediators, and restrict the tumor-induced angiogenesis. SCFAs also sustain the integrity of basement membranes and modulate the intestinal pH. CRC patients have lower concentrations of SCFAs than healthy individuals. Increasing the production of SCFAs through the manipulation of the gut microbiota could constitute an important therapeutic strategy towards CRC due to their antitumorigenic effect and ability of modulating tumor microenvironment.

## 1. Colorectal Cancer Therapeutic Challenges

Colorectal cancer (CRC) is one of the most common malignant tumors worldwide being the third in terms of incidence but the second in terms of mortality [1]. Its incidence is related with lifestyle factors, such as a low-fibre diet, smoking, lack of exercise, and obesity [2,3]. Despite the evolution on the research for new therapeutics, the management of CRC still poses several therapeutic challenges. Some of these challenges include late diagnosis since CRC often presents with no symptoms in its early stages making it difficult to diagnose until it has progressed to later stages, which can reduce the effectiveness of the treatment. The resistance to chemotherapy also constitutes a difficulty for an effective treatment, along with the high probability for the development of a metastatic disease. Importantly, the side effects associated with CRC treatments can impact the quality of life of patients [4].

Despite all these challenges, there are several therapeutic options currently being clinically used to treat CRC. However, it is important to understand that CRC comprises two different types of tumors, namely colon cancer and rectal cancer [5]. In fact, they are often grouped together due to the several common symptoms (such as bleeding, pain, and changes in stool), but anatomically the colon is approximately five feet long in the large intestine, while the rectum is the last five to six inches of the colon that connects to the anus [5]. Taking this into account, depending on the diagnosis and stage of the disease, the first line of treatment for both colon and rectal cancers consists of achieving the complete removal of the tumor and metastases by surgical intervention [6]. However, when the cancer is diagnosed at an advanced stage with metastases, the surgical control becomes difficult and, for those patients, the best option is to shrink the tumor, inhibiting the tumor spread and growth by chemotherapy [6]. This strategy might also be applied before or after surgery as adjuvant treatment to maximally reduce and stabilize the tumor [6].

Regarding the use of neoadjuvant radiotherapy, it is an approach using high-energy rays (such as x-rays) or particles to destroy tumor cells and it is currently used to treat rectal cancer (RC), not colon cancer [7]. In fact, it has become the standard treatment for stage II/III rectal cancer patients to help reduce the size of a tumor or kill cancer cells that have spread [7]. This treatment can also be applied after the resection to destroy remaining cancer cells and used mainly in stage II/III RC patients who have not received preoperative radiotherapy [7].

Considering the cytotoxic chemotherapeutic drugs, they act by targeting cells that proliferate rapidly [8]. The current chemotherapy for CRC comprises single-agent therapy, mainly fluoropyrimidine (5-FU)-based (FOL), and multiple-agent regimens containing one or several drugs, including oxaliplatin (OX), irinotecan (IRI), and capecitabine (CAP) [6]. The antimetabolite drug 5-FU exerts its anticancer effects through the inhibition of thymidylate synthase and incorporation of its metabolites into RNA and DNA [9]. OX is a platinum-based chemotherapy drug that damages the DNA in cancer cells, which slows their growth and division [10]. It is often used in combination with 5-FU and leucovorin [10]. IRI (Campto) is also used in combination with 5-FU and leucovorin and works by inhibiting the DNA topoisomerase, which slows their growth and division [11]. CAP (Xeloda) was approved as an oral prodrug of 5-FU for use against CRC, being converted to 5-FU after absorption across the digestive tract [12]. Considering all the available chemotherapeutic drugs, the combined therapy regimens FOLFOX (5-FU+OX), FOLFIRI (5-FU+IRI), XELOX or CAPOX (CAP+OX), and CAPIRI (CAP+IRI) constitute the mainstream approaches in first-line treatment [6].

The increased knowledge about the hallmarks of cancer allowed the development of targeted therapies, which can work on tumor cells by directly affecting specific features, such as cell proliferation, differentiation, migration, and even the tumor microenvironment [13]. Currently, in addition to 5-FU-, OXI-, and/or IRI-containing chemotherapy regimens, immunotherapy and targeted therapy regimens are becoming an increasingly important option for the treatment of metastatic CRC. The combination of the chemotherapy with or without the biological therapies, such as angiogenesis inhibitors (bevacizumab (Avastin) or ramucirumab (Cyramza)), epidermal growth factor receptor (EGFR) inhibitors (panitumumab (Vectibix) or cetuximab (Erbitux)), or immunotherapy, may be considered [14].

The use of EGFR inhibitors, such as panitumumab or cetuximab, is a possibility preferable for patients with left-sided tumors with wild-type KRAS, NRAS, or BRAF genes [14]. Unfortunately, 40% of metastatic CRCs harbour KRAS mutations, which often lead to constitutive activation of the mitogen-activated protein kinase (MAPK) pathway and are associated with resistance to anti-EGFR drugs [15]. NRAS and BRAF mutations, although constituting a lower percentage of the total CRC cases (approximately 4 and 10%, respectively), are also associated with less effective responses to these types of drugs [15,16]. Considering the anti-angiogenic agents, bevacizumab is a humanized monoclonal antibody that targets vascular endothelial growth factor (VEGF), having a significant role in the treatment of metastatic CRC [17]. The biologic drug ramucirumab can also be administered as an angiogenesis inhibitor in metastatic CRC patients, and like bevacizumab, it is a fully humanized immunoglobulin G1 monoclonal antibody that binds with high affinity to the VEGFR-2 extracellular domain, blocking all VEGF ligands from binding to this target [18,19]. This drug is normally used with FOLFIRI to treat metastatic CRC when the cancer continues to progress during or after other treatments. Another available biological drug is regorafenib (Stivarga) that acts as a multi-kinase inhibitor, namely through the inactivation of angiogenic and oncogenic kinases, such as VEGF 1–3, fibroblast growth factor receptor 1, EGFR, RAF, and tyrosine-protein kinase [18,20]. It is designed to treat patients with metastatic colon cancer whose cancer has continued to advance after approved standard therapies [18,20].

There is still another possibility for CRC patients with unresectable, metastatic tumors, the single-agent immunotherapy. The PD-1 inhibitors nivolumab (Opdivo) and pembrolizumab (Keytruda) can be administrated in patients with advanced or metastatic tumors with deficient mismatch repair (dMMR) or microsatellite instability-high (MSI-H) [14].

Despite the reported advances in CRC therapy, it is important to disseminate preventive measures, including maintenance of a healthy body weight, physical activity, minimization of red and processed meat and alcohol consumption, and avoidance of smoking [2]. Specific changes in human intestinal microbiota (dysbiosis) associated to sporadic CRC have also been described [2,21]. In fact, it has been observed in CRC patients an enrichment of detrimental bacterial communities (pro-inflammatory opportunistic pathogens) and a reduction in commensal bacterial species (butyrate-producing bacteria). These trillions of microorganisms residing within the gut can modulate the CRC susceptibility and progression through mechanisms spanning from activating inflammation and/or DNA damage and by producing metabolites (the so called microbe-associated molecular pattern) involved not only in tumor progression or suppression but also to therapy’s response [22].

## 2. The Tumor Microenvironment

The tumor microenvironment is a complex and continuously evolving entity, which consists of tumor cells, tumor stromal cells, including stromal fibroblasts, endothelial cells (ECs), and immune cells, and the non-cellular components of the extracellular matrix (ECM) [23,24]. The interaction of the tumor cells with their microenvironment is dynamic and bidirectional and includes cell–cell contacts, or cell–free contacts (involving ECM), and the mediators that enable these contacts [24]. The mediators can be soluble factors (chemokines, cytokines, and growth factors) or those that facilitate horizontal genetic/biomaterial transfer, namely cell-free DNA (cfDNA), apoptotic bodies, circulating tumor cells (CTCs), and exosomes [24]. Through such complex crosstalks, the tumor microenvironment components are significantly involved in cancer progression and metastasis either by promoting or inhibiting the process [24,25].

The microbiota is also an important element of the tumor microenvironment, and Hanahan suggests that polymorphic variation in microbiomes of the intestine and other organs, as well as the tumor microbiome, may constitute a distinctive enabling characteristic for the acquisition of hallmark capabilities [13]. Microbiota play a dual role of promoting or inhibiting cancer progression, and its metabolites can be important modulators of the tumor microenvironment [26]. As discussed in the next sections, some metabolites, such as SCFAs, contribute to the regulation of inflammation, proliferation, cellular energetics and metabolism, gene expression, and cell death [13,26].

The CRC tumor microenvironment, in particular, which includes the intestinal microbiota, is not only closely related to the growth and development of cancer but also affects the treatment and prognosis of the patients [27]. It can limit the efficacy of therapeutic agents through high interstitial pressure, fibrosis, and the degradation of the therapeutic agents by enzymatic activity [28]. Moreover, the tumor microenvironment is a special niche in terms of acidity, hypoxia, and ischemia, and its components can modulate tumor progression by stimulating angiogenesis, suppressing apoptosis, or inducing immunodepression [28].

It has been demonstrated that the tumor microenvironment plays an important role in both cancer progression and tumor-induced cachexia through the production of multiple pro-cachectic factors [29]. This condition constitutes a multifactorial syndrome in patients with advanced cancer characterized by weight loss via skeletal-muscle and adipose-tissue atrophy, catabolic activity, and systemic inflammation [29]. In CRC, cachexia affects around 50–61% of patients and is correlated with functional impairment, reduced therapeutic responsiveness, and poor prognosis, being a major cause of death in cancer patients [29]. It has been already reported the role of different components of the CRC tumor microenvironment in the production of procachetic agents, namely pro-inflammatory cytokines (e.g., TNF-α, IL-6, and IL-1) and certain chemokines [29]. However, it is important to find strategies to target the tumor microenvironment in order to modulate the production of these procachetic agents and, in this way, enhance the therapy response and prognosis.

Due to the importance of the tumor microenvironment for the efficacy of the different available therapeutic agents, strategies for its modulation are being investigated in the cancer immunotherapy field. It is already known that the limited retention time is a problem for the approaches using drugs that target the tumor microenvironment. To overcome this limitation and allow the delivery of drugs in the tumor microenvironment, nanoparticles with unique physical properties and design have been developed [30]. The possibility of creating an efficient drug delivery system with different ligands that could specifically target components in the tumor microenvironment, namely dendritic cells, macrophages, fibroblasts, tumor vasculature, and the hypoxic state, has been studied [30]. These systems could also influence the aberrant structures and functions of the tumor microenvironment and in this way reducing the development of drug resistance and improving the response to chemotherapy. However, there are still several challenges to overcome in order to translate the “tumor-microenvironment-targeting nanoparticles” to clinical practice, one of them being the limited knowledge about the immune network of the different types of cancer, as well as the heterogeneity between the tumor microenvironment of different patients [30]. New insights about therapy strategies that target/modulate the tumor microenvironment are continuously emerging. In order to improve the knowledge about the different factors affecting the tumor microenvironment, this review aims to understand the effects of SCFAs on the tumor microenvironment constituents, particularly in the context of CRC.

## 3. Short-Chain Fatty Acids in the Human Colon

The intestinal microbiota, strongly modulated by the diet, plays an important role in maintaining host health, including protection against pathogens, maturation of the immune system, degradation of toxic substances, digestion of complex carbohydrates, and production of short-chain fatty acids (SCFAs) [2]. SCFAs are mainly produced from dairy diet-derived microbiota, and they are the major products of bacterial fermentation of undigested dietary fibres and starch. They were shown to be able to influence the progress of several diseases, in particular inflammatory bowel disease (IBD), diabetes, atherosclerosis, and CRC [31]. Gut microbiota-derived SCFAs, in particular acetate, butyrate, and propionate, can affect the energetic metabolism, enhance barrier function, reduce low-grade inflammation, and suppress tumor progression [32]. The ratio of these metabolites in the colonic lumen is approximately 60% acetate, 25% propionate, and 15% butyrate, butyrate being the primary energy source for colonocytes [2]. The level of SCFAs in faecal samples has been associated with some diseases, including cancer, being already reported that CRC patients have a decreased faecal SCFAs concentration compared to healthy individuals [2,33]. Understanding how the increase on SCFAs in the colon might be beneficial not only by their antitumorigenic properties but also by modulating the tumor microenvironment might have an important impact in CRC therapy and will be explored here.

As mentioned above, the concentrations of SCFAs in CRC patients are known to be low [2,33]. This raised the question whether increasing the levels of these microbiota metabolites would be a good strategy for prevention and/or better responsiveness to anticancer treatment. This could be achieved by optimizing their production with specific nutritional diets, specifically with the intake of fibres and appropriate probiotics [2]. Faecal microbiota transplantation is another possible approach [32].

Following their production, SCFA’s transport into the intestinal epithelial cells is mediated by transporter proteins, such as monocarboxylate transporter 1 (MCT1), which is coupled to H+ transport, the sodium-coupled monocarboxylate transporter 1 (SMCT1), and aquaporins [2,34,35]. SCFAs that are not metabolized by the colonocytes are transported through portal circulation and can also reach systemic circulation directly through the inferior vena cava [34]. These gut microbiota-derived metabolites can be taken up by other organs where they act as substrates or signalling molecules that regulate several cellular processes and systemic effects [34]. These effects are mediated mainly by two pathways: the inhibition of histone deacetylases (HDACs) and the activation of cell surface G-protein-coupled receptors (GPRs), namely GPR41 (also known as free fatty acid receptor 3, FFAR3), GPR43 (FFAR2), and GPR109A [2,34].

Several studies have demonstrated the antitumorigenic properties of SCFAs [35,36,37,38,39,40,41,42,43], and their effects on malignant cells have been already reviewed [2,32,34]. Among the three mentioned SCFAs, butyrate has been the most studied [2]. It reduces survival and induces cell death in CRC cells through several mechanisms, depending on its intracellular concentration [2]. Its protective effects against human colon cancer cells involve inhibition of cell differentiation, promotion of cell-cycle arrest and apoptosis, modulation of histone acetylation, and decrease of pro-inflammatory factors with an increase in the anti-inflammatory cytokine interleukin 10 (IL-10) [2].

Propionate induces typical signs of apoptosis in human CRC cell lines with a loss of mitochondrial membrane potential, generation of reactive oxygen species (ROS), cytochrome c release, caspase-3-processing, and nuclear chromatin condensation [2]. It can induce autophagy, which serves as an adaptive strategy to retard mitochondria-mediated cell death in CRC cells [2]. Propionate, like butyrate, also acts as an inhibitor of cell growth and as an inducer of acetylation in CRC cells [2].

Despite being the least studied, acetate also affects CRC cells [2]. It was shown to decrease viability and to induce typical signs of apoptosis, including loss of mitochondrial membrane potential, generation of ROS, caspase-3 processing, and nuclear chromatin condensation in the colon adenocarcinoma cell line HT-29 [2]. Our group has shown that acetate treatment in CRC cells decreases cell proliferation and induces apoptosis, in a process characterized by DNA fragmentation, caspase-3 activation, and phosphatidylserine exposure to the outer leaflet of the plasma membrane with the appearance of a sub-G1 population [41]. Moreover, acetate induces lysosomal membrane permeabilization with cathepsin D (CatD) release to the cytosol, which has an anti-apoptotic role in acetate-induced apoptosis [41,42]. Our group has also described that acetate induces an alteration in the energetic metabolism through the modulation of monocarboxylate transporters expression (MCTSs) [41,42].

Despite being known that each SCFA plays a role in several biological processes, it is also important to understand the combined effects of the three SCFAs, since colon cells are exposed to these compounds simultaneously [44]. Recently, our group reported that all three SCFAs, alone or combined at the physiological proportions found in the human colon (60 acetate: 15 butyrate: 25 propionate), revealed to have a selective and anticancer effect by inhibiting colony formation and cell proliferation, increasing apoptosis, disturbing the energetic metabolism, inducing lysosomal membrane permeabilization, and decreasing cytosolic pH [44]. Additionally, this study showed for the first time that SCFAs are specific towards colon cancer cells, showing promising therapeutic effects [44]. All the current evidence concerning the effects of acetate, butyrate, and propionate on CRC cells, alone or in combination, clearly supports the potential use of SCFAs in cancer prevention and treatment [2]. However, we must as well take into consideration their influence on components of the tumor microenvironment.

## 4. Short-Chain Fatty Acids and the Tumor Microenvironment

As mentioned before, the different tumor microenvironment components are significantly involved in cancer progression and metastasis, being already used as a target in immunotherapy approaches, however, with low success rates for CRC. On the other hand, SCFA’s therapeutic potential has been proven by the scientific community, showing promising results in blocking CRC cells proliferation and inducing cell death by apoptosis. In this regard, understanding the influence of SCFAs on the different constituents of the tumor microenvironment could constitute an important issue, but to the best of our knowledge, there is still a lack of reviews on this matter. To overcome this, we performed an original, up to date critical literature review on the current knowledge about the effects of microbiota-derived short-chain fatty acids (acetate, butyrate, and propionate) on the different components of the tumor microenvironment, namely, immune cells (lymphocytes, macrophages, dendritic cells, and neutrophils), endothelial cells, pH, and extracellular matrix.

### 4.1. Role of Short-Chain Fatty Acids in the Regulation of Immune Cells

**Figure 1:**
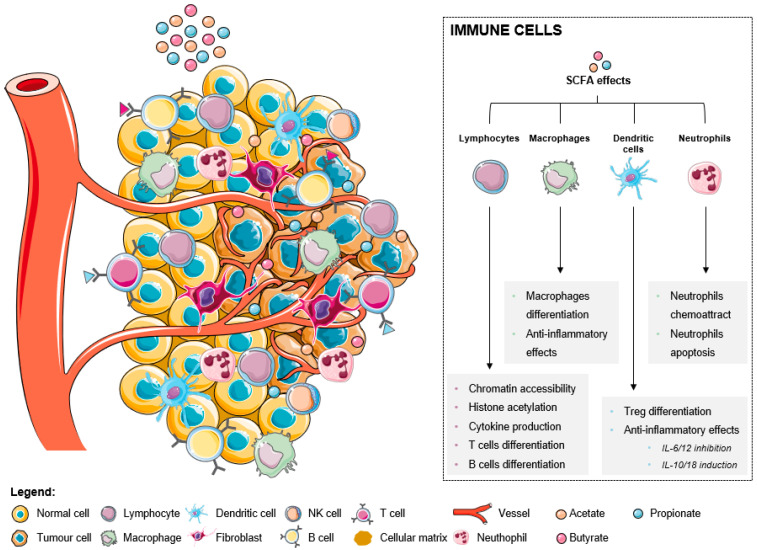
The effects of short-chain fatty acids in the tumor microenvironment-associated immune cells.

Immune cells are critical components of the tumor microenvironment that can act both as suppressors of tumor initiation and progression, as well as promoters of proliferation, infiltration, and metastasis [45]. Broadly, they fall into two categories: adaptive immune cells, such as T and B lymphocytes, and innate immune cells, including macrophages, mast cells, neutrophils, dendritic cells (DCs), myeloid derived suppressor cells (MDSCs), and natural killer (NK) cells [13]. Both pro- and antitumorigenic roles of each cell type in the tumor microenvironment and treatment responses have been thoroughly described [23,24,25,27,45]. A good prognosis in CRC has been attributed to infiltration by Th1 cells, M1 macrophages, mature DCs, and NK cells, while the presence of M2 macrophages, MDSCs, Th17, and B cells has been associated with a poor outcome [46]. The increase of SCFA levels affect immune cells in distinct ways, inducing several effects in different immune cells (as summarized in Figure 1). In lymphocytes, there is an increased chromatin accessibility, histone acetylation, cytokine production, and T and B cells differentiation. In fact, it was already described that ILC3s, T cells, and B cells in the intestine are the primary targets of regulation by SCFAs. This happens because the levels of SCFAs are highest in the gut, where SCFAs support the activity of these lymphocytes to promote balanced intestinal immunity and immune tolerance [47]. Recent data suggest that the SCFAs play a role in the metabolism of effector T cells. These metabolites are able to regulate cytokine expression and T cell function through HDAC inhibition and by providing acetyl groups for acetyl-CoA, which is a donor substrate for HATs mediating histone acetylation [48].

Macrophage differentiation is also induced with associated anti-inflammatory effects. It was recently reported that butyrate directs the differentiation of homeostatic macrophages that possess strong antimicrobial activity through the inhibition of HDAC3 by regulating their metabolic and transcriptional program [49]. In addition, it was proven that acetate, butyrate, and propionate affects macrophage (specifically M2) differentiation partly through G-protein-coupled receptor 43 (GPR43) activation and/or HDAC inhibition [50].

SCFAs also promote anti-inflammatory effects through the inhibition of IL-6 and IL-12, the induction of IL-10 and IL-16 in dendritic cells, and consequent Treg differentiation. It was reported that the dendritic cells’ expression of amphiregulin, a molecule of the epidermal growth factor (EGF) family, which is a critical regulator of cell proliferation and tissue repair, depends on butyrate through its interaction with GPR43 [51]. Comparing the three metabolites, acetate only exerts negligible effects, while both butyrate and propionate are described as being able to strongly modulate gene expression in both immature and mature human monocyte-derived dendritic cells [52].

Regarding neutrophils, these cells are attracted to the tumor microenvironment in response to SCFA treatment, consequently suffering apoptosis. A recent study demonstrated for the first time that SCFAs induce the formation of neutrophil extracellular traps (NET) when neutrophils are exposed to the intestinal physiological concentrations of these acids [53]. The process of NET formation involves the release of chromatin structures in the form of an extracellular network containing nuclear or mitochondrial DNA with the objective of using these structures being to trap pathogens and prevent their spread in the organism [53]. Importantly, a study in which acetate was administrated to animals revealed that this metabolite was effective in controlling inflammatory response by inducing caspase-dependent apoptosis of neutrophils that accounted for the resolution of inflammation [54]. Resolution of neutrophilic inflammation was associated with decreased NF-κB activity and enhanced production of anti-inflammatory mediators, including IL-10, TGF-β, and annexin A1 [54].

#### 4.1.1. Effect of Short-Chain Fatty Acids in Lymphocyte Populations

Within the tumor microenvironment there are several distinct populations of lymphocytes [23]. Cytotoxic T cells (CD8+) detect abnormal tumor antigens expressed on cancer cells and target them for destruction [23]. They also suppress angiogenesis through the secretion of interferon gamma (IFN-γ) [23]. CD4+ T cells differentiate into a variety of subtypes, including helper T cells, and thus coordinate a wide range of immune responses within the context of the tumor microenvironment [23]. Regulatory T cells (Tregs) are ubiquitous and promote tumor development and progression by dampening antitumor immune responses [23]. Additionally, Tregs directly support the survival of cancer cells through the secretion of growth factors and indirectly through interaction with stromal cells [23]. B cells recognize tumor antigens and produce specific antibodies against the tumor with the cooperation of helper T cells, decreasing tumor progression [21].

Recent studies have suggested the ability of SCFAs to affect the tumor immune response [21,55,56,57]. T cells in the tumor microenvironment must compete with tumor cells for available glucose and other nutrients, which limits T cell activity and favors tumor progression [56]. Effector T cells and tumor cells share many metabolic features, such as engaging Warburg metabolism (aerobic glycolysis) [56]. In a hypoxic or nutrient-deprived state, acetate (and other SCFAs) is an important alternative carbon source for cancer cells to support survival and proliferation through its conversion to acetyl-CoA by acetyl-CoA synthetases (ACSS) [58]. Acetate also affects immune cell function [56]. For instance, a systemic increase in acetate induced by infection is required for optimal memory CD8+ T cell function through a mechanism involving increased glyceraldehyde-3-phosphate dehydrogenase acetylation and enhanced glycolysis [59]. Given this, researchers studied whether acetate could correct cytokine production in glucose-restricted T cells and, ultimately, T cells in the tumor microenvironment [56]. It was shown that prolonged glucose restriction contributes to T cell hyporesponsiveness, marked by defects in IFN-γ production, and that administration of acetate promotes chromatin accessibility, histone acetylation, and cytokine production (Figure 1) in glucose-restricted T cells in an ACSS2-dependent manner [56]. However, more work is required to determine the relevance of the use of acetate by T cells in a variety of in vivo settings.

Other studies have suggested that butyrate can affect the tumor immune response via promoting T cell differentiation (Figure 1) into effector and Treg cells [57]. In many cancer therapies, less infiltration or dysfunction of CD8+ T cells in the tumor microenvironment results in poor clinical outcomes [55]. Butyrate can modulate antitumor CD8+ T cell responses through the inhibition of differentiation 2-dependent IL-12 signaling, suggesting that this SCFA can promote anticancer immunity to sufficiently improve therapeutic efficacy [55]. Most of the studies were conducted in mice, and whether butyrate could also influence antitumor CD8+ T cell immunity in humans remains to be determined, as well as whether different doses of butyrate could differentially regulate many cells in the tumor microenvironment. Additionally, butyrate downregulates CRC-related adverse events of indoleamine 2,3-dioxygenase 1 (IDO1) expression via a signal transducer and activator of a transcription 1 (STAT1)-dependent way or as the histone deacetylase (HDAC) inhibitor [32]. IDO1 can activate β-catenin signaling to promote cancer cell proliferation and colon tumorigenesis [32].

Cytotoxic T lymphocyte-associated antigen-4 (CTLA-4) is a protein found on T cells. When CTLA-4 is bound to another protein called B7, it helps keep T cells from killing other cells, including cancer cells [60]. Some anticancer drugs, called immune checkpoint inhibitors, are used to block CTLA-4 [60]. Despite their beneficial effects, systemic butyrate and propionate appear to limit the antitumor activity of CTLA-4 blockade in hosts with cancer and are associated with a higher proportion of Tregs [61].

B cells, or B lymphocytes, participate in immune regulation mainly by immunoglobulin production, antigen presentation, and secretion of cytokines [27]. Typically, they are concentrated at the margin of the tumors and they are commonly found in lymph nodes near the tumor microenvironment [23]. Compared to T cells, relatively few infiltrating B cells are found in the tumor microenvironment, but they are important in the formation of “tertiary lymphoid structures” that allow close association between T and B cells [23]. The antitumorigenic roles of B cells include antigen-presentation to T cells, antitumor antibody production, and secretion of cytokines, such as IFN-γ, which promote cytotoxic immune responses [23]. On the other hand, B cells can have pro-tumor effects [23]. For example, regulatory B cells promote tumor aggression through the production of cytokines, including IL-10 and transforming growth factor-beta (TGF-β), that promote immune suppressive phenotypes in macrophages, neutrophils, and cytotoxic T cells [23]. A recent study highlighted the diverse phenotypes of B cells in the CRC microenvironment, which can explain the formerly described contradictory effects of B cells on tumors [62].

Microbiota-derived SCFAs support intestinal B cell differentiation (Figure 1) and antibody production by inhibiting HDAC activity leading to increased histone acetylation and gene expression of multiple genes associated with B cell function [63,64]. A study using mice fed with special diets or drinking water containing an SCFA mixture for 4 weeks revealed that SCFAs effectively increase cellular metabolism in B cells, which provides energy and building blocks to support B cell activation, differentiation, and antibody production [64]. Furthermore, butyrate and propionate restrict normal B cell intrinsic functions, including immunoglobulin class switching and somatic hypermutation [63]. Thus, microbiota-derived SCFAs have potent immunomodulatory effects on immune cells in the host that actively maintain homeostasis and dampen inflammation in the intestine [63].

#### 4.1.2. Short-Chain Fatty Acids Modulate Macrophages

Macrophages are critical elements of the innate immune system, modulating immune responses through pathogen phagocytosis and antigen presentation, and are also critical in wound healing and tissue repair [23]. In most types of solid cancers, the infiltration of tumor-associated macrophages (TAMs) is usually linked with a poor survival and enhanced metastasis [65]. However, in CRC the infiltration of TAMs is linked with better prognosis [65]. TAMs can be subdivided into two categories based on their activation status, M1 (classically activated) or M2 (alternatively activated), which in most cases are considered pro-tumorigenic [65]. M1 TAMs are driven by IFN-γ, whereas alternative M2 TAMs are driven by IL-4 and IL-13 [65]. The tumor microenvironment promotes the M2 phenotype through hypoxia and the secretion of cytokines to support tumor growth and progression [23]. M2 TAMs produce high levels of reactive oxygen free radicals, promote DNA damage and genomic instability, tumor infiltration and metastasis, participate in the digestion and reconstruction of ECM, and inhibit antitumor immunity [66]. M2 TAMs marker expression is a poor prognostic factor in CRC [21]. Studies haves shown that SCFAs, specifically butyrate, can modulate the immune response of colonic macrophages through the inhibition of HDAC [49,67]. It directs the differentiation of homeostatic macrophages (Figure 1) that possess strong antimicrobial activity and play an important role in preventing the dissemination of bacteria beyond the intestinal barrier [49]. Exposure of mouse macrophages to butyrate downregulates pro-inflammatory mediators (Figure 1), restoring intestinal immune homeostasis [67]. This data has implications for the prevention and therapy of disorders that are associated with intestinal inflammation. On the other hand, butyrate enhances M2 macrophage polarization and function [68].

#### 4.1.3. Dendritic Cells Modulation by Short-Chain Fatty Acids

Dendritic cells (DCs) are considered the most professional antigen presenting cells (APCs) [65]. They recognize, capture, and present antigens to T cells at secondary lymphoid organs, linking the gap between adaptive and innate immunity [23]. Ideally, DCs within the tumor microenvironment surround tumor associated antigens and migrate towards the draining lymph nodes, where they stimulate T cell-mediated responses [65]. DCs are inherently programmed to have an antitumorigenic function in the body, but the tumor microenvironment can co-opt them to support tumor progression [23]. In a suppressive environment that hinders their maturation, they become tolerogenic or regulatory DCs, which promote tumor cell survival [65].

Nastasi and collaborators observed that GPR41 and GPR109A are both expressed by human DCs, indicating that both these receptors may be important for the SCFAs induced signal transduction [52]. Their study revealed that butyrate and propionate play a crucial role in modulating immune responses in human mature DCs, showing a strong anti-inflammatory effect (Figure 1) by inhibiting the expression of lipopolysaccharide-induced cytokines, such as IL-6 and IL-12p40 [52]. Moreover, the activation of GPR43 and GPR109A in intestinal epithelial cells and DCs promotes the secretion of IL-18 and IL-10, respectively [34]. IL-10 promotes the differentiation and proliferation of Tregs (Figure 1) that together with IL-18 protect against conditions leading to colonic inflammation and CRC [34]. Wu and co-workers proposed that the activation of GPR43 by acetate on DCs leads to the expression of aldehyde dehydrogenase [69]. This enzyme converts vitamin A into retinoic acid, which induces B cell immunoglobulin A production, although this mechanism is still under debate [34,69].

#### 4.1.4. Short-Chain Fatty Acids Impact on Neutrophil Functions

Neutrophils are effector cells of the innate immune system, accounting for 50–70% of human circulating leukocytes and provide the first line of defense against many pathogens [23,27]. Like other immune cells, they can both promote and suppress tumor formation and progression [25]. As a tumor begins to grow, neutrophils are recruited to the tumor microenvironment and promote inflammation through the release of cytokines and ROS that stimulate tumor cell apoptosis [23]. Later in tumor development, neutrophils promote tumor growth through modification of the extracellular matrix, releasing vascular endothelial growth factor (VEGF) and producing matrix metalloprotease (MMP)-9 to stimulate angiogenesis and, ultimately, tumor progression and local invasion [23].

Several neutrophil functions are modulated by SCFAs. Both in vivo and in vitro evidence suggests that, in a non-inflammatory condition, SCFAs act as neutrophil chemoattractants (Figure 1) through activation of GPR43 [70]. It has been described that butyrate and acetate increase ROS production by neutrophils and phagocytosis in a GPR43-dependent manner [70]. However, some researchers demonstrated that SCFAs have no effect or even inhibit ROS production and phagocytosis by neutrophils depending on the type of stimuli, neutrophil source, state of activation of the cells, concentration of the SCFAs, and type of assay used [70]. In addition, propionate and butyrate induce apoptosis in both activated and non-activated neutrophils (Figure 1) through the activation of caspases, independent of the activation of SCFA receptors [70]. SCFAs can also modify neutrophil recruitment by their ability to regulate the production of inflammatory mediators and neutrophil-chemoattractants [71]. The relevance of the effects depicted needs to be further investigated in vivo, especially in the context of CRC.

### 4.2. Role of Short-Chain Fatty Acids in the Regulation of Endothelial Cells

Vascular endothelium is a thin monolayer of endothelial cells (ECs) that help to orchestrate the formation of blood vessels [23]. It separates circulating blood from tissues, delivers water and nutrients, maintains metabolic homeostasis, carries immune cells, and participates in the formation of new blood vessels (angiogenesis) [23]. Angiogenesis is crucial for cancer progression by supplying oxygen and nutrients while removing toxic metabolites and also provides a conduit for tumor cell dissemination and metastasis [25]. Hypoxia-inducible factors initiate vessel sprouting by instructing ECs to secrete proangiogenic factors, such as platelet-derived growth factor (PDGF), epidermal growth factor (EGF), and vascular endothelial growth factor (VEGF) [23]. Tumor associated ECs also produce growth factor receptors, such as VEGF and EGF receptors, to enhance angiogenesis [25]. In an autocrine and paracrine process, VEGF stimulates the migration of ECs to form new blood vessel lumens, and then ECs secrete proteins to form new basement membranes [23]. Blood vessels in the tumor microenvironment often fail to achieve the final stages of maturation and lack proper cell-to-cell connections, resulting in leaky vasculature and enabling cancer cells to transverse it [23].

**Figure 2:**
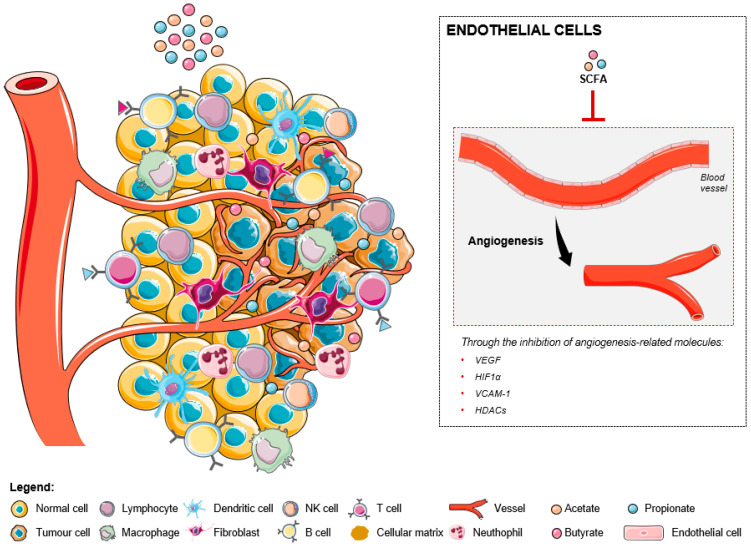
The effects of short-chain fatty acids in the tumor microenvironment-associated endothelial cells. The increase of SCFA levels inhibit the expression levels of several angiogenesis-related molecules, namely, VEGF, HIF1α, VCAM-1, and HDACs, resulting in the inhibition of the formation of new blood vessels.

Studies in two different CRC cell lines [72,73] showed that butyrate was able to modulate the expression of two important angiogenesis-related molecules: VEGF, the most potent angiogenic factor, and hypoxia-inducible factor (HIF)-1α, the main transcription activator of the VEGF gene [72]. These results suggested that butyrate could inhibit tumor-induced angiogenesis in human CRC (Figure 2). Conversely, the effects of the others microbiota-derived SCFAs were not evaluated. A recent study determined that SCFAs could significantly inhibit IL-6 and IL-8 production, as well as vascular cell adhesion molecule 1 (VCAM-1) expression on activated endothelial cells, by activation of GPR41 and GPR43 and inhibition of HDACs [74]. Further studies are required to evaluate the effects of SCFAs in tumor-associated ECs. Nevertheless, it should be noted that IL-6 and IL-8 are involved in the development of CRC [75,76] and that VCAM-1 (upregulated in human CRC tissues) promotes invasion and metastasis via activating the epithelial-mesenchymal transition (EMT) program [77].

### 4.3. Impact of Short-Chain Fatty Acids on pH

**Figure 3:**
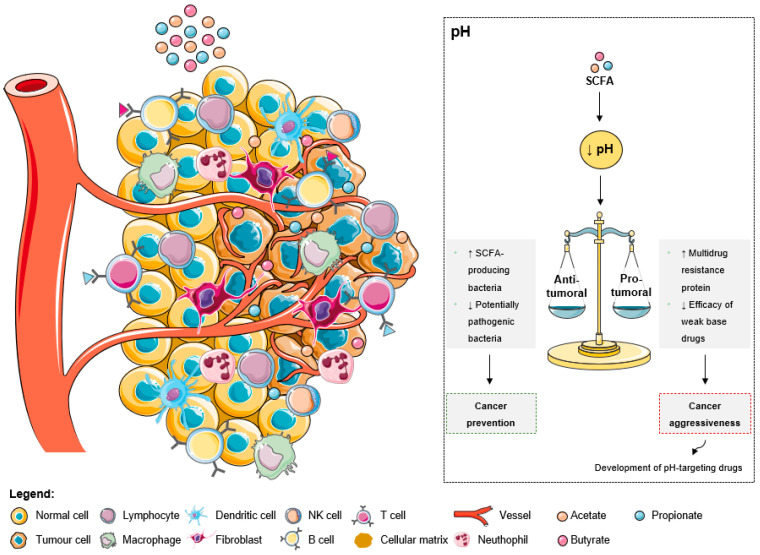
The effects of short-chain fatty acids in the tumor microenvironment pH. The increase of SCFA levels leads to a decrease of the extracellular pH. This acidic pattern contributes to cancer prevention due to the anti-tumoral effects, namely the increase of SCFA-producing bacteria and the decrease of pathogenic microbes. Additionally, the low extracellular pH could promote aggressiveness due to the increase of multidrug resistant proteins and the decrease of weak base drugs’ efficacy. However, these consequences are being overcome with the development of pH-targeting drugs.

As explained by the Warburg effect, tumors present a constitutive and persistent upregulation of glycolysis, which results in chronic acidification [78]. To adapt and survive in adverse conditions, including local hypoxia and poor vasculature perfusion, cancer cells upregulate membrane pH regulators as a self-defensive strategy [78]. This keeps an intracellular pH ranging from neutral to slightly alkaline due to an increased secretion of protons to the tumor microenvironment, leading to its acidification [78]. The acidic tumor microenvironment has been associated with certain key features of cancer aggressiveness (Figure 3), including invasion, evasion from the immune system, increased angiogenesis, and resistance to therapy, making it an attractive target for therapy [78]. Additionally, a low extracellular pH also contributes to drug resistance [78,79,80]. The acidic pH gradient generated between intra- and extracellular space affects the distribution and uptake of weak base chemotherapeutic drugs [79]. These drugs become charged (ionized form), which compromises their transport across the plasma membrane and their further cytoplasmic accumulation leading to a lower cytotoxicity [78]. Moreover, low external pH has been shown to dramatically increase the expression of the multidrug resistance protein in human colon carcinoma cell lines [80]. To overcome these drawbacks, researchers are considering the development of drugs specific to target acidic environments [81].

The intestinal pH also impacts bacterial growth and activity [82]. A lower pH may promote the growth of SCFA-producing bacteria, while inhibiting the growth of potentially pathogenic bacteria sensitive to low pH, and, in turn, high SCFAs concentrations may lower intestinal pH at the same time (Figure 3) [82,83]. Due to the beneficial health effects of SCFAs and the strong interplay between their concentrations and pH, a lower fecal pH could indicate improved gut health [82]. In fact, studies revealed a significant increase in fecal pH in patients with CRC compared to healthy individuals [2]. Additionally, a colonic environment with lower SCFAs levels and higher pH was considered at high risk of developing CRC [84]. Mucosal cells adapt to these conditions and acquire epigenetic and genetic changes to survive, predisposing to tumorigenesis [84].

As shown by Lan and co-workers [85], extracellular pH influences the mode of cell death triggered by propionibacteria-produced SCFAs in CRC cells [2]. At pH 7.7, acetate and propionate decrease proliferation and induce cell cycle arrest in G2/M, followed by a sequence of cellular events characteristic of apoptosis, while necrosis was induced at pH 5.5 [2]. This apoptosis–necrosis switch induced by SCFAs at lower pH might be of importance for cancer therapy, especially for the treatment of solid tumors known to be related to an acidic microenvironment [85].

In summary, it appears that a more acidic colonic luminal pH is associated with both beneficial and deleterious effects on colonocytes according to the status of colonocytes (healthy or neoplasic) [80]. Increased SCFA production and concomitant lower pH may promote a healthy colon and prevent the development of CRC [82].

### 4.4. Impact of Short-Chain Fatty Acids in Extracellular Matrix

The ECM is composed of glycoproteins, collagen, elastin, proteoglycans and other macromolecules, which support and connect tissues and maintain normal physiological functions [27]. Compared with normal tissue, the ECM structure of tumor tissue is disordered [27]. ECM proteins can be produced by many stromal cell types and tumor cells, however, cancer-associated fibroblasts (CAFs) are the main source for synthesis, secretion, assembly, and modification of the ECM composition and organization [24].

Accumulation of significant amounts of collagen, together with a high percentage of fibroblast infiltration, result in desmoplasia, which is strongly linked to poor patient prognosis and resistance to therapy [23,27]. In general, ECM abnormalities relieve the behavioural regulation of stromal cells and promote tumor-related angiogenesis and inflammation, resulting in resistance to immunotherapy in the tumor microenvironment [27]. Each individual ECM protein contributes to CRC progression and metastasis in distinct ways, but the overall amount of ECM protein deposition contributes to the stiffness of the tumor microenvironment [25]. Correspondingly, increased ECM stiffness is a hallmark of CRC progression and metastasis [25]. On the other hand, the ECM can hamper cancer progression as well, acting as a natural barrier for tumor cell proliferation, differentiation, and metastasis [25].

**Figure 4:**
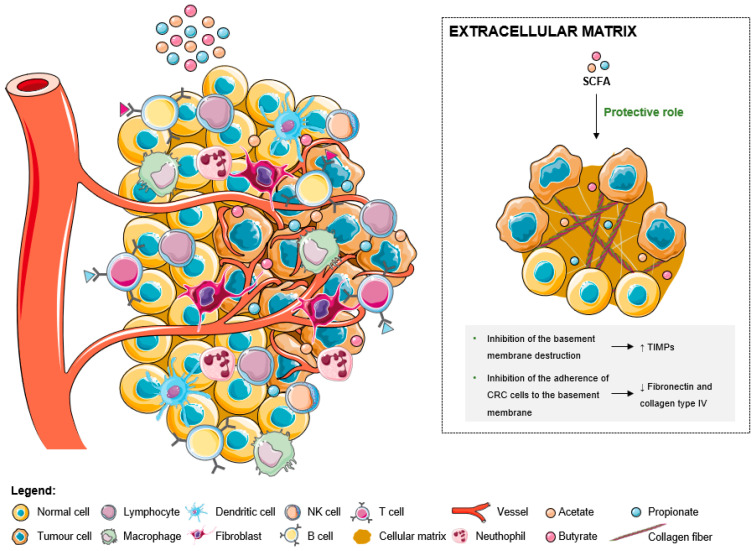
The effects of short-chain fatty acids in the tumor microenvironment extracellular matrix. SCFA play a protective role against the basement membrane destruction by the overexpression of TIMPs. Additionally, the reduction of the levels of fibronectin and collagen type IV leads to the inhibition of the CRC cells’ adherence to the basement membrane.

Matrix metalloproteases (MMPs) are degrading enzymes that break down ECM proteins and are critical in remodeling ECM to promote tumor progression and metastasis [23]. Colon tumor cells can induce the secretion of MMP2 and MMP9 by stromal cells via direct contact or paracrine regulation [86]. Tissue inhibitors of matrix metalloproteinase (TIMPs) also regulate ECM remodeling through the inhibition of MMPs, retarding tumorigenicity, metastasis, and the invasive cell phenotype induced by MMPs [86,87]. SCFAs, particularly butyrate, can inhibit the destruction of the basement membrane through the stimulation of the TIMPs (Figure 4) [87,88]. They also inhibit the adherence of colon cancer cells to the basement membrane by reducing fibronectins or type IV collagen levels, resulting in the inhibition of cancer invasion (Figure 4) [89]. Even so, TIMP-1-expressing cells are more resistant to chemotherapy than are TIMP-1 gene-deficient, and in CRC patients, high levels of TIMP-1 in tumor tissue and plasma are strongly associated with shorter survival time [86].

## 5. Therapeutic Implications of the Tumor Microenvironment Modulation by Short-Chain Fatty Acids

Given the critical functions of the tumor microenvironment, from both microorganisms and the host, in CRC progression and metastasis and the accumulating knowledge on this subject, new insights about CRC therapy by targeting tumor microenvironment are emerging [25]. One of the most common therapies for CRC is to target VEGF and prevent EC-mediated angiogenesis [25]. However, this method has significant adverse side effects and limited benefit to the patients because it targets both tumor-associated and normal ECs [25]. Aside from targeting or remodeling cellular components in the tumor microenvironment, manipulating non-cellular components could have therapeutic potential as well [25].

Nowadays, research on a great variety of targets and approaches is being carried out [27,90]. These include antiangiogenic therapy, adoptive cell therapy, immune checkpoint inhibitors, cancer vaccine and oncolytic virus therapy, and tumor-derived exosomes therapy [27]. Several studies suggest that SCFAs can contribute to anticancer therapeutic efficacy [91,92,93]. Approximately 50% of patients suffer from gastrointestinal mucositis after pelvic or abdominal radiation treatment, and the incidence is higher in patients undergoing concurrent chemotherapy [91]. Besides the impact of gut microbiota on the response to diverse forms of cancer therapy, tumor treatments may in turn affect the microbiota (that is, induce dysbiosis), which can consequently aggravate the inflammation triggered by radiation and chemical reagents [91]. SCFAs might contribute to restoring bacterial homeostasis, attenuating inflammation, maintaining the barrier function, promoting antitumor effects, and mucosal repair after cancer treatments [91]. Furthermore, it was shown that the chemotherapeutical efficacy of 5-FU on CRC cells was promoted by the combined treatment of butyrate and 5-FU, with lower DNA synthesis efficiency and higher apoptotic cell ratios [92]. It was also demonstrated that butyrate suppresses the proliferation of three-dimensional CRC organoids and enhances radiation-induced cell death in CRC organoids [93]. However, butyrate does not increase radiation-induced cell death after irradiation in normal organoids [93]. Accordingly, it may enhance the efficacy of radiotherapy while protecting the normal mucosa [93]. These findings support the idea that adjusting food intake, regulating gut bacteria, and subsequently altering the concentration of SCFAs is a promising approach in CRC treatment.

At the clinical level, further studies are still needed to confirm the use of SCFA as adjuvants; however, all existing information indicates that these compounds have a high therapeutic potential. In this way, increasing the SCFAs concentration in the colon, either through the adoption of specific dietary patterns (such as a dairy rich diet) or even through the ingestion of dietary supplements such as pro-, pre- or symbiotics, may result in a favorable response to the therapy [94].

## 6. Conclusions

Considering the high incidence of CRC and the drawbacks associated with conventional therapies, interest in developing new strategies for cancer prevention and treatment has increased in the last years [2,65]. Particularly, the evidence regarding the connection between CRC and gut dysbiosis led researchers to consider the modulation of intestinal microbiota as an attractive alternative and/or adjuvant approach.

A large number of studies have enlightened the antitumorigenic properties of colon microbiota-derived SCFAs and their effects in CRC cells [2,6,16,18,19,20,21,22,23,24,25]. Additionally, as described in this review, they can modulate the tumor microenvironment in many distinct ways. SCFAs antitumorigenic effects include promoting differentiation, chromatin accessibility, histone acetylation, and cytokine production in tumor infiltrating lymphocytes [56], directing macrophage differentiation, downregulating pro-inflammatory mediators [49], restricting tumor-induced angiogenesis [72], inhibiting the destruction of basement membranes [88], lowering intestinal pH, and improving gut health [82]. However, some pro-tumorigenic results have also been described. High blood butyrate and prothispionate levels are associated with resistance to CTLA-4 blockade and a higher proportion of Treg cells [32]. Butyrate enhances M2 macrophage polarization and function, and these macrophage phenotype is associated with poor prognosis in CRC [21,68].

Current evidence supports the potential use of SCFAs or nutraceuticals that increase their production in the colon for prevention and treatment of CRC. The present study had some limitations since this was a comprehensive narrative review in which we considered the effects of the SCFAs in the main components of the tumor microenvironment. However, it is important to refer that there are some additional elements that may play a role but are still under study. Some of the mechanisms underlying SCFAs modulatory effects in CRC tumor microenvironment components need to be clarified. Similarly, further studies are required to understand how these different mechanisms influence each other in the complexity of the tumor microenvironment in CRC. Summing up, the manipulation of the gut microbiota in order to increase the production of SCFAs might constitute a clinically relevant CRC therapeutic strategy due to SCFA’s dual effect as antitumorigenic as well as modulators of tumor microenvironment.

## Data Availability

Data sharing not applicable.
